# Galactose: A Versatile Vector Unveiling the Potentials in Drug Delivery, Diagnostics, and Theranostics

**DOI:** 10.3390/ph17030308

**Published:** 2024-02-27

**Authors:** Chiara Battisegola, Chiara Billi, Maria Cristina Molaro, Marica Erminia Schiano, Maria Nieddu, Mariacristina Failla, Elisabetta Marini, Stefania Albrizio, Federica Sodano, Maria Grazia Rimoli

**Affiliations:** 1 Department of Pharmacy, “Federico II” University of Naples, 80131 Naples, Italy; c.battisegola@studenti.unina.it (C.B.); chiara.billi@unina.it (C.B.); mariacristina.molaro@unina.it (M.C.M.); maricaerminia.schiano@unina.it (M.E.S.); salbrizi@unina.it (S.A.); rimoli@unina.it (M.G.R.); 2 Department of Medicine, Surgery and Pharmacy, University of Sassari, 07100 Sassari, Italy; marvi@uniss.it; 3 Department of Drug Science and Technology, University of Turin, 10125 Turin, Italy; mariacristina.failla@unito.it (M.F.); elisabetta.marini@unito.it (E.M.)

**Keywords:** D-galactose, prodrugs, drug delivery, diagnostics, theranostics

## Abstract

D-galactose, a simple natural compound, has been investigated as a powerful scaffold for drug delivery, diagnostics, and theranostics due to its distinctive properties and interactions with specific cell receptors. In the field of drug delivery, galactose functions as a ligand to selectively target cells expressing galactose receptors, such as hepatocytes, macrophages, and specific cancer cells. The direct attachment of galactose to the main drug or to drug-loaded nanoparticles or liposomes enhances cellular uptake, thereby improving drug delivery to the intended target cells. Galactose has also been found to be useful in diagnostics. Specifically, diagnostic tests based on galactose, such as the galactose elimination capacity test, are utilized to evaluate liver function and assess liver disease as well as hepatic functional reserve. Additionally, galactose-based theranostic agents can be designed by combining drug delivery and diagnostic capabilities. This review is an update of our previous review concerning the broad spectrum of possibilities for exploiting D-galactose as a vector for prodrug design and the synthetic strategies that allow its realization, jointly in diagnostics and theranostics, to highlight the versatility of this interesting vector.

## 1. Introduction

Carbohydrates are one of the most important and abundant classes of biomolecules [1] as they are major components of genetic material and are the main vital source of energy [2]. They play essential roles in cellular and intracellular interactions thanks to their action as signaling molecules and their presence on cell surface receptors [3]. These functions explain the potential of carbohydrates for use as drug delivery [4,5,6], diagnostic, and theranostic agents [4,7,8,9].

D-galactose (Gal), a monosaccharide sugar, has demonstrated its utility as a ligand in various applications. It has been investigated as a vector for drug delivery, diagnostics, and theranostics due to its distinctive properties and interactions with specific cell receptors.

In the realm of drug delivery, Gal serves as a ligand for targeting specific cells that express Gal receptors, including hepatocytes, macrophages, and certain cancer cells. By attaching Gal directly to the parent drug or to the drug-loaded nanoparticles or liposomes, cellular uptake can be enhanced, leading to improved drug delivery to the desired target cells. This approach has the potential to enhance drug efficacy while minimizing adverse effects [4,5,6].

Gal has also been found to be useful in diagnostics since it can be used as a marker for certain diseases and conditions, as its presence or absence can be an indicator of specific metabolic processes within the body. Diagnostic tools such as blood tests and imaging techniques that exploit Gal have been developed to aid in the diagnosis of various illnesses. Galactose-based tests, such as the Gal elimination capacity test, have been employed in assessing liver function to evaluate liver disease and hepatic functional reserve. These tests measure the liver’s ability to metabolize Gal, providing valuable insights into liver health and disease progression [4].

Another intriguing area is theranostics, which integrates therapeutic and diagnostic functions into a single system. Galactose-based theranostic agents can be designed by combining drug delivery and diagnostic capabilities. For instance, by incorporating imaging agents like fluorescent dyes or radioactive tracers into galactose-conjugated nanoparticles, it becomes possible to track the distribution of the therapeutic payload and monitor treatment response simultaneously [7].

**Figure 1:**
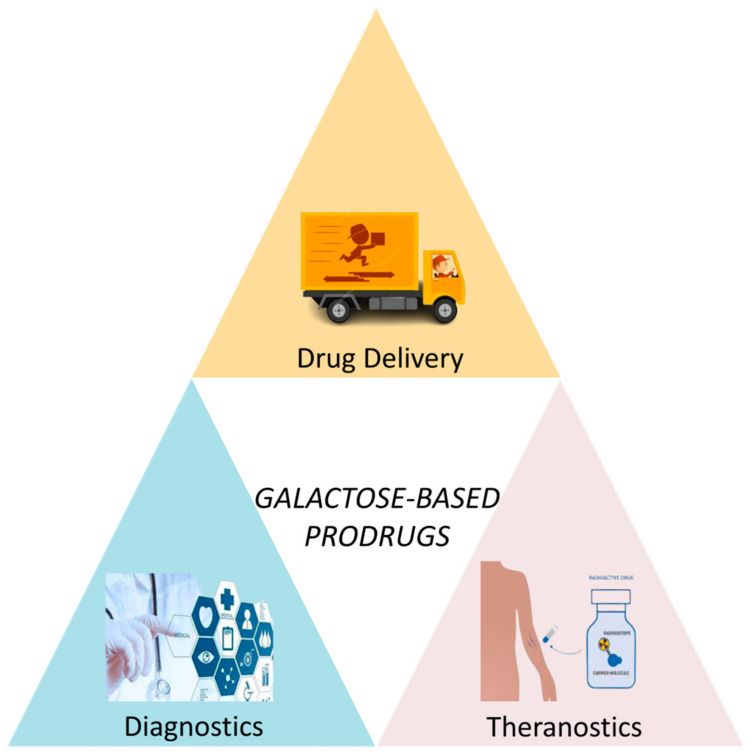
The role of Gal in various fields of medicinal chemistry.

As illustrated schematically in Figure 1, this review will thoroughly explore the role of Gal in these three main fields, i.e., drug delivery, diagnostics, and theranostics, with the aim of highlighting the versatility of this interesting vector. Our attention is focused on representative examples reported in the literature in the years 2012–2023.

## 2. Galactose for Drug Delivery

Gal moiety can act as a ligand that specifically targets cells expressing Gal receptors, such as hepatocytes, macrophages, and certain cancer cells. There are a lot of examples that demonstrate the potential of Gal as a drug delivery system for various therapeutic applications, leveraging its targeting capabilities and biocompatibility. However, it is important to note that the success of galactose-based drug delivery systems depends on factors such as the choice of carrier, the conjugation strategy, and the specific receptor expression profile of the target cell. The outcomes of studies have shown diverse findings depending not only on the specific linkage established between the drug and the hydroxyl group but also the position of Gal hydroxyl insertion can make a difference [10].

Gal can be conjugated directly to the parent drug or to drug carriers, such as nanoparticles or liposomes, through covalent or non-covalent linkages. These drug-loaded carriers can be designed to release the drug at the target site, improving therapeutic efficacy and minimizing adverse effects. Through manipulation of these parameters, researchers have not only achieved selective pharmacological targeting of brain, liver, and cancerous cells but also enhanced cellular permeability and improved the pharmacokinetic profile of the parent drugs. The examples of galactose conjugated to drug carriers and galactose conjugated directly to parent drugs considered in this review have been shown schematically in Table 1 and Table 2, respectively.

### 2.1. Galactose Conjugated to Drug Carriers

The approach of Gal conjugation to a drug carrier has been extensively explored in the context of liver disorders, which encompass a range of conditions such as viral hepatitis, fibrosis, cirrhosis, and cancer. Targeted drug delivery to hepatocytes is crucial for an effective treatment, as it allows for higher drug concentrations at the site of action while minimizing off-target effects and reducing systemic toxicity.

In the liver, both parenchymal and Kupffer cells possess receptors on their plasma membranes that specifically bind to and internalize ligands carrying terminal Gal residues [10,36]. The well-studied asialoglycoprotein receptor (ASGP-R) is present on parenchymal cells and serves as the most extensively characterized model for receptor-mediated endocytosis through the clathrin-coated pit pathway. Notably, the quantity of ASGP-Rs in human-isolated hepatocytes from livers with histological alterations is higher than that present in normal livers.

**Scheme 1:**
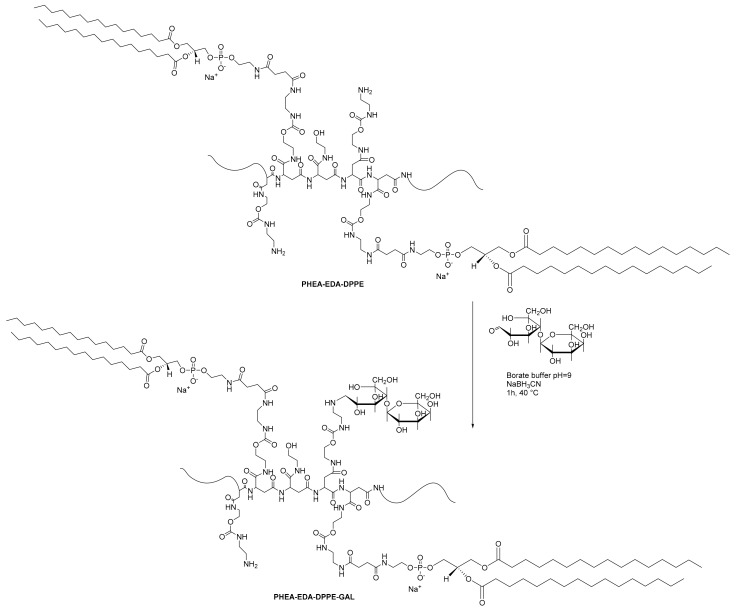
Synthesis of PHEA-EDA-DPPE-GAL copolymer.

Craparo et al. have reported the preparation of liver-targeted nanoparticles that can potentially carry a ribavirin (RBV) prodrug to hepatocytes for the treatment of the Hepatitis C virus [11]. RBV, an anti-viral ribonucleoside analog, is one of the essential drugs in some combination chemotherapies, and it exhibits anti-viral activity in hepatocytes infected with the hepatitis C virus. However, repeated administration of RBV frequently induces severe hemolytic anemia because it extensively accumulates in erythrocytes [37]. As reported in Scheme 1, the nanoparticles were prepared starting from a galactosylated phospholipid-polyaminoacid conjugate, which was obtained by a chemical reaction of α,β-poly(*N*-2-hydroxyethyl) (2-aminoethylcarbamate)-D,L-aspartamide (PHEA-EDA) and 1,2-dipalmitoyl-sn-glycero-3-phosphoethanolamine-*N*-(succinyl) sodium salt (DPPE), and subsequently with lactose to yield PHEA-EDA-DPPE-GAL copolymer. To enhance the entrapment into obtained nanostructures, a hydrophobic RBV prodrug, RBV tripalmitate, was synthesized, and its capability to release RBV in the presence of adequate enzymatic activity was demonstrated. RBV tripalmitate-loaded nanoparticles were obtained using the dialysis method, and they showed a spherical shape and nanometric size. In vitro experiments showed the absence of hemolytic activity of RBV tripalmitate-loaded PHEA-EDA-DPPE-GAL nanoparticles. Their specificity toward HepG2, a liver cancer cell line, was found by using a competitive inhibition assay in the presence of free Gal and assessing nanoparticle uptake in the presence of free Gal and/or non-galactosylated nanoparticles.

**Scheme 2:**
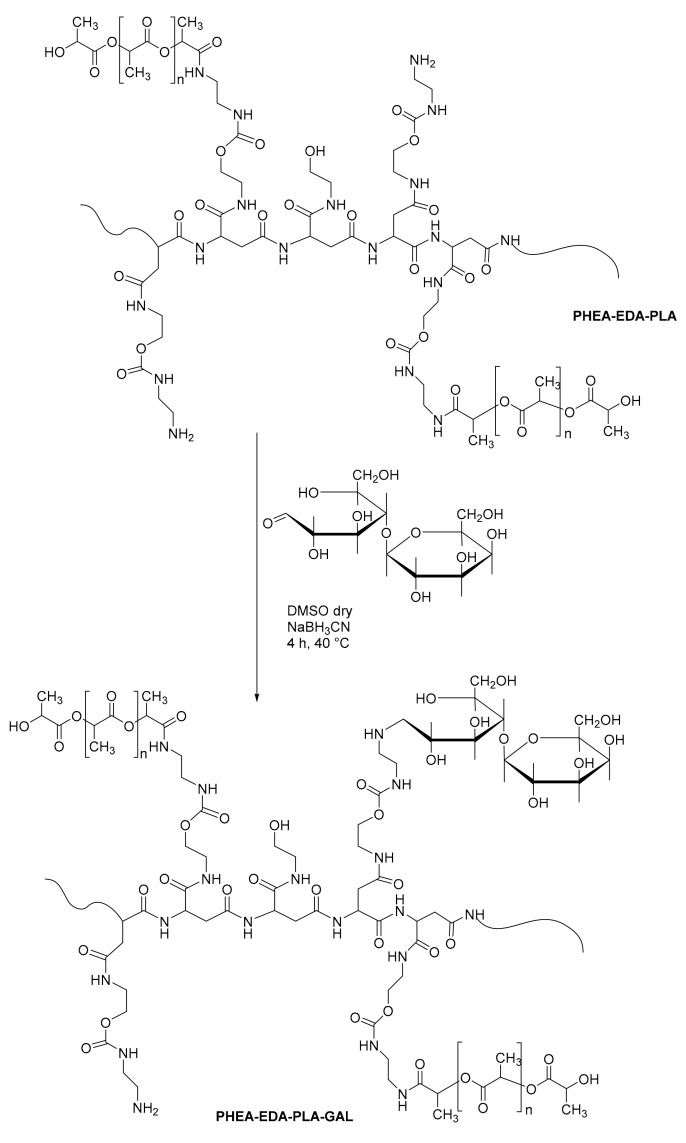
Synthesis of PHEA-EDA-PLA-GAL copolymer.

In another work, the same authors have developed galactosylated micelles that can potentially carry the prodrug RBV tripalmitate to hepatocytes [12]. As reported in Scheme 2, the micelles were prepared using a galactosylated polylactide–polyaminoacid conjugate, which was obtained by a chemical reaction of PHEA-EDA with polylactic acid (PLA) and subsequent reaction with lactose, resulting in PHEA-EDA-PLA-GAL copolymer. The micelles were designed to exploit the presence of carbohydrate receptors, specifically ASGP-R, which are present on hepatocytes. By in vitro experiments, the specificity of RBV tripalmitate-loaded PHEA-EDA-PLA-GAL micelles toward HepG2 was demonstrated by using a competitive inhibition assay in the presence of free Gal.

In this system, chemically modified RBV (RBV tripalmitate) was used as a hydrophobic prodrug because, in this form, the molecules could be easily encapsulated in the loci of solid particles through hydrophobic interactions. However, RBV tripalmitate would also need to be converted to RBV in vivo to exhibit its activity.

Kaneko et al. have focused on ribavirin monophosphate (RMP) as a drug [13]. RBV undergoes intracellular phosphorylation by cellular kinases to form a monophosphate, a diphosphate, and a triphosphate, all of which have anti-viral activities [38]. This suggests that RBV directly functions as an active drug without chemical changes in vivo. Additionally, the accumulation of RBV in erythrocytes may be suppressed due to the difficulty of phosphorylated nucleosides to penetrate cell membranes [39].

**Figure 2:**
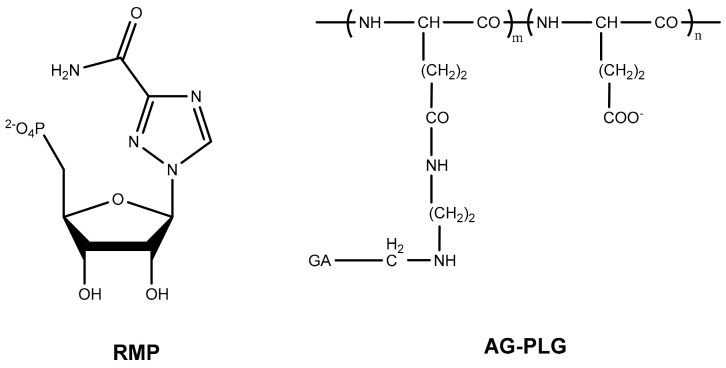
Chemical structures of RMP and AG-PLG.

As a carrier for RMP, the authors have chosen biodegradable solid nanoparticles (NPs), successfully prepared from a mixture of poly(D,L-lactic acid) with terminal amino group(s) and a conjugate (AG-PLG) consisting of arabinogalactan (AG) and poly(L-glutamic acid) (PLG), as depicted in Figure 2.

The carrier was able to specifically deliver the drug to the liver without significant drug release in the circulation and, consequently, no RBV accumulation in erythrocytes. Liver targeting was due to the polymer content of AG, which is a polysaccharide containing Gal side chains. Gal-decorated NPs specifically bind ASGP-R on hepatocytes, inducing endocytosis and release of cargo inside the liver cells.

**Scheme 3:**
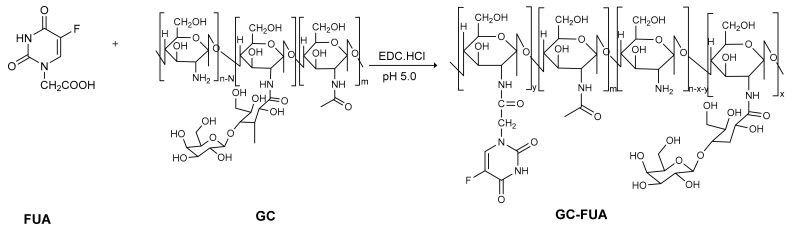
Synthesis of GC-FUA.

Liver-targeted anti-cancer therapy is another interesting field for Gal application. Indeed, hepatocellular carcinoma is one of the most malignant tumors, and chemotherapy is still the main treatment for advanced and recurrent hepatocellular carcinoma, with all the related disadvantages, such as low bioavailability, poor selectivity, intrinsic or acquired drug resistance, effects on healthy cells, and immunosuppression [40]. To overcome these drawbacks, Yu et al. studied a novel type of macromolecular prodrug (GC–FUA), *N*-galactosylated chitosan (GC)−5-fluorouracil acetic acid (FUA) conjugate-based nanoparticles, designed and synthesized as a carrier for hepatocellular carcinoma drug delivery as can be seen in Scheme 3 [41]. FUA is an anti-cancer drug commonly used in the treatment of various cancers, including liver cancer.

GC was used as a carrier for 5-FUA since it is known to have a high affinity for the ASGP-R expressed on hepatocytes. In this work, GC–FUA nanoparticles were produced by an ionic crosslinking method based on the modified ionic gelation of tripolyphosphate with GC–FUA. The average particle size of GC–FUA NPs was 160.1 nm, and their drug-loading content was 21.22 ± 2.7%. An in vitro drug-release study showed that GC–FUA NPs displayed a sustained-release profile compared to FUA-loaded GC nanoparticles. All the results suggest that GC–FUA NPs may have great potential for anti-liver cancer applications.

A few years later, the same macromolecular prodrug GC-FUA-based nanoparticles was investigated for its in vitro cytotoxicity and cellular uptake, in vivo pharmacokinetic, and tissue distribution [14]. The biocompatibility of GC-FUA-NPs was screened by bovine serum albumin (BSA) adsorption test and hemolysis activity examination in vitro. Cytotoxicity and cellular uptake study in HepG2 and A549 cells demonstrated that compared to free 5-FUA, GC-FUA NPs play a great function in killing cancer cells, thanks to the cell endocytosis mediated by ASGP-R overexpressed on the cell surface. Pharmacokinetics study further illustrated that the drug-loaded nanoparticles have a much longer half-time than free 5-FUA in blood circulation in Sprague–Dawley rats. The obtained data confirmed that GC-FUA NPs are a very promising drug delivery system for the efficient treatment of hepatocellular carcinoma.

In a follow-up study carried out by Ning et al., employing the same macromolecular prodrug approach, the researchers have made an elegant combination of gene therapy and nanocarrier chemotherapy [15]. The issues addressed were the targeted delivery of liver-specific microRNA 122 (miRNA-122) and 5-FUA. To achieve this, they used a technique called electrostatic condensation, which refers to the process of aggregating or condensing charged particles or molecules through electrostatic interactions. In this work, miRNA-122 was condensed with GC-FUA, and the rationale behind this approach is that miRNA-122 plays a crucial role in liver function and can act as a potential therapeutic target for liver-related diseases. By employing electrostatic condensation, the researchers aimed to improve the delivery efficiency and specificity of miRNA-122 and 5-FUA to liver cells. Additionally, the receptor-mediated targeting strategy ensured that the drug would preferentially accumulate in the liver, reducing off-target effects and enhancing therapeutic efficacy. The codelivery of miRNA-122 and 5-FUA using the GC-FU system allows the synergistic effect of both therapies. Indeed, miRNA-122, by specifically targeting liver cells, can modulate gene expression and potentially enhance the sensitivity of liver cancer cells to 5-FUA treatment. This approach holds promise for improving the therapeutic outcomes for liver-related diseases, particularly liver cancer. The effectiveness of delivery was assessed in vitro and in vivo. Specifically, the biocompatibility of GC-FU/miRNA-122 nanoparticles was examined through analyses of hemolysis activity, BSA absorption, and cell viability in normal liver cells (L02 cells) and endothelial cells. The developed codelivery systems inhibited the proliferation of hepatocellular carcinoma (HCC) cells, induced in HepG2 cells apoptosis and the downregulation of two target genes of the miRNA, ADAM17 and Bcl-2, as well as greater anti-tumor efficacy in vivo (BALB/c nude mice) [15].

**Scheme 4:**
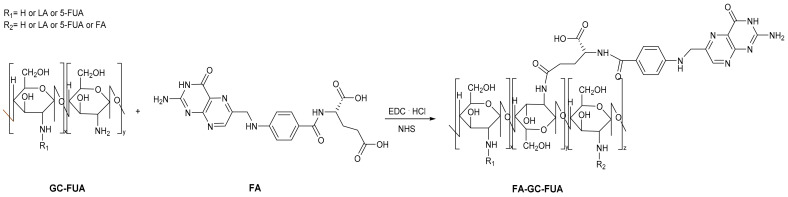
Synthesis of FA-GC-FUA.

The same research group later reported another study introducing a simple yet efficient strategy for the development of dual-targeting ligand-functionalized nanoparticles, aiming to achieve precise therapy for HCC and potential clinical translation [16]. To accomplish this, they incorporated folic acid (FA) as a hydrophobic and targeting component into the hydrophilic macromolecular prodrug GC-FUA. This combination resulted in the formulation FA-GC-FUA, which exhibited self-assembly into nanoparticles driven by the solubility variation of FA and GC-FUA, eliminating the need for previously used physical crosslinking methods (Scheme 4).

The resulting FA-GC-FUA NPs were designed to selectively target two specific receptors found on the surface of HCC cells: ASGP-Rs and folate receptors (FRs). The FA and lactobionic acid (LA) residues exposed on the nanoparticle surface enabled the binding to FRs and ASGP-Rs, respectively. This dual-targeting approach maximized the efficiency of targeting HCC cells while minimizing non-specific uptake by normal hepatocytes, as demonstrated by in vitro and in vivo experiments. In in vitro competition cytotoxicity and cellular uptake studies, it was observed that the cellular uptake mechanism for FA-GC-FUA NPs in SMMC-7721 cells was synergistically mediated by both folate receptors and ASGP-Rs. However, this synergistic mechanism is not observed in A549 cells. This lack of identification introduces some disagreement between the results of the cell apoptosis assay and the MTT-assessed in vitro anti-tumor effect. In vivo animal studies provide confirmation of the significantly improved therapeutic efficacy and safety of FA-GC-FUA NPs compared to free 5-FUA. These results demonstrate the high potential of the FA-GC-FUA formulation for clinical translations.

Finally, Wang et al. focused on the development of hepatocyte-targeting anti-tumor prodrugs mediated by ASGP-R and triggered by glutathione [17]. In their work, the authors highlight the use of targeting ligands coupled with therapeutic drugs as a strategy to improve tissue selectivity and reduce side effects. ASGP-R, which is overexpressed in hepatocytes, is identified as a potential target for the specific delivery of drugs to hepatocytes. Glutathione, which is overexpressed in tumor cells, is proposed as a trigger for the release of drug payloads in tumor tissues.

**Figure 3:**
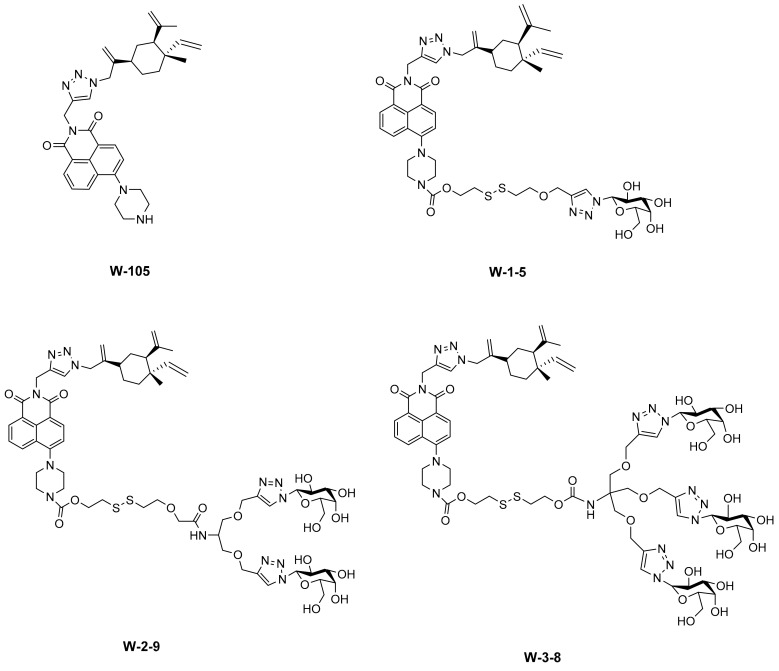
Chemical structures of W-105, W-1-5, W-2-9, and W-3-8.

The synthesis of hepatocyte-targeted prodrugs was carried out by using a β-elemene derivative W-105 served as the anti-tumor pharmacophore drug payload, Gal as the hepatocyte-targeting ligand, and the disulfide responsive to the tumor cells (high concentration of glutathione) served as the linking group. To fully take advantage of the galactose-recognition cluster effect toward ASGP-R, the parent compound was modified by one, two, and three molecules of Gal to afford mono-, bi-, and tridentate-galactose prodrugs, respectively, as shown in Figure 3 (W-105, W-1-5, W-2-9, and W-3-8). The hepatocyte-targeting capacities of these compounds were found to be in the order W-105 (parent compound) < W-1-5 (monodentate-galactose) < W-2-9 (bidentate-galactose) < W-3-8 (tridentate-galactose). This order is attributed to the excellent affinity of the Gal ligand to ASGP-R and the galactose-cluster recognition effect. The prodrug W-3-8 exhibited good in vivo anti-tumor activity and low toxic side effects. Liquid chromatography–mass spectrometry assays revealed that these prodrugs could release the anti-tumor pharmacophore in the presence of high glutathione amounts (mimicking the condition of the tumor cell) and maintain the low-toxic structures with normal levels of glutathione (mimicking the condition of the normal cell). In vitro evaluations of the hepatocyte-targeting capabilities of these prodrugs were presented. The synthesized prodrugs have potential in the treatment of liver cancer due to their hepatocyte-targeting properties and the triggered release mechanism in tumor tissues.

In the search for innovative approaches for the treatment of reactive oxygen species-mediated diseases in the liver, Sakai et al. successfully created PEG (polyethylene glycol)-Sulfo-BSA-GAL, a targeting agent for hepatic and intrahepatic delivery of an H_2_S prodrug [18]. This agent, PEG-Sulfo-BSA-GAL, exhibited selective accumulation in liver nonparenchymal cells and parenchymal cells. Importantly, PEG-Sulfo-BSA-GAL demonstrated minimal H_2_S release in the bloodstream but selectively released H_2_S in RAW264.7 cells (mouse macrophage-like cells) and HepG2 cells. H_2_S is a naturally occurring gas transmitter that plays a critical role in various biological functions, including vasodilation, anti-oxidation, anti-inflammation, and cytoprotection [42]. Reduced H_2_S production contributes to heightened intrahepatic resistance in cirrhotic livers. To address this, the researchers developed two types of sulfo-albumins: Sulfo-BSA-Suc and PEG-Sulfo-BSA-GAL. These sulfo-albumins are capable of selectively releasing H_2_S intracellularly. For the biodistribution experiments using mice, each Sulfo-BSA was radiolabeled with ^111^In (indio), and it was observed that up to 80% of ^111^In-labeled Sulfo-BSA-Suc and PEG-Sulfo-BSA-GAL accumulated rapidly in the liver, within 30 min after intravenous injection. Additionally, ^111^In-labeled Sulfo-BSA-Suc primarily accumulated in liver nonparenchymal cells (endothelial cells and Kupffer cells), while PEG-Sulfo-BSA-GAL predominantly accumulated in liver parenchymal cells (hepatocytes). These findings have significant implications for the targeted delivery of H_2_S for the treatment of liver diseases associated with reactive oxygen species.

**Scheme 5:**
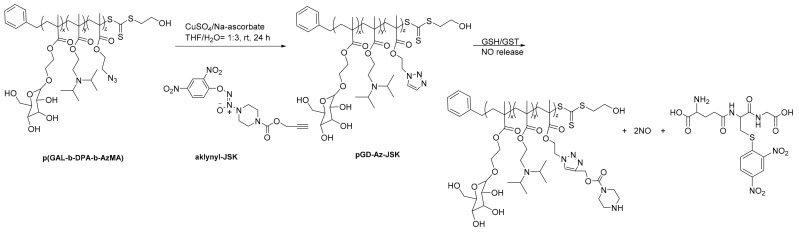
Synthesis of pGD-Az-JSK.

In another paper, Zhang et al. describe the development of a nanoparticle-based delivery system for the co-delivery of nitric oxide (NO) and doxorubicin (DOX) for cancer treatment [19]. The nanoparticles were designed to be pH-responsive, self-assembled, and targeted to the Gal receptor. NO prodrug 2-(2,4-dinitrophenyl) 1-[4-(propargyloxycarbonyl)piperazin-1-yl]diazen-1,2-diolate (alkynyl-JSK) was chemically conjugated to an amphiphilic block copolymer, termed p(GAL-b-DPA-b-AzMA), through a click reaction to form NO prodrug nanoparticles (pGD-Az-JSK) (Scheme 5). The nanoparticles exhibited high NO capacity, good stability, and a sustained NO release pattern with unique glutathione/glutathione S-transferase (GSH/GST) activated NO-releasing kinetics. Gal receptor targeting capability of the nanoparticles was demonstrated in vitro using HepG2 cells. The nanoparticles also showed superior cytotoxicity to HepG2 cells when combined with DOX chemotherapy due to their rapid acid-triggered DOX release and sustained NO release. The study suggests that these multifunctional nanoparticles can serve as an efficient NO and chemotherapeutic agent delivery platform, holding great promise in cancer combinatorial treatment.

**Figure 4:**
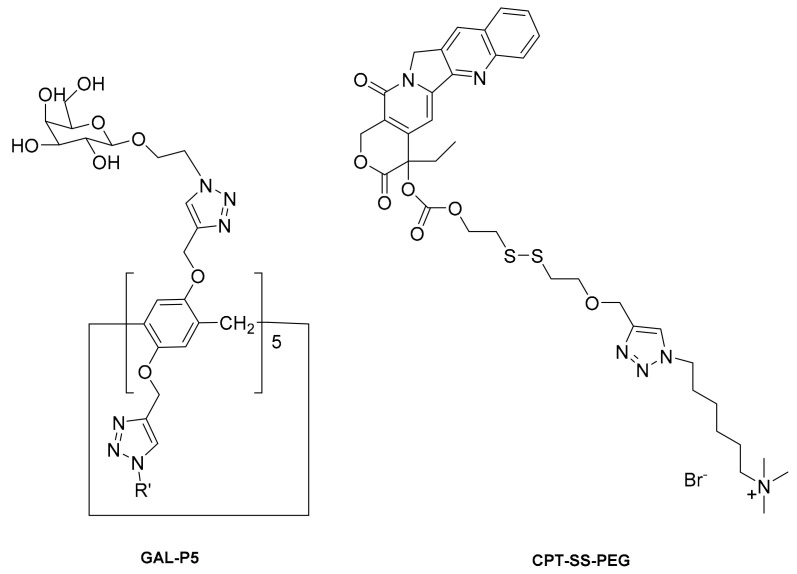
Chemical structures of GAL-P5 and CPT-SS-PEG.

Liu et al. describe the development of a supramolecular nanoparticle-based delivery system for the targeted delivery of camptothecin (CPT) prodrug for cancer treatment [20]. The nanoparticles were designed to be GSH-responsive, self-assembled, and targeted to the Gal receptor. The nanoparticles were constructed by the host–guest interaction between β-D-GAL-modified pillar[5]arene (GAL-P5) and CPT prodrug (CPT-SS-PEG), whose structures are reported in Figure 4. The nanoparticles exhibited high drug loading capacity, good stability, and a sustained drug release pattern with unique GSH-responsive drug-releasing kinetics. Gal receptor targeting capability of the nanoparticles was demonstrated in vitro using HepG2 cells. The nanoparticles also showed superior cytotoxicity to HepG2 cells when compared to free CPT due to their rapid GSH-triggered drug release and targeted delivery. The study suggests that these multifunctional nanoparticles can also serve as an efficient, targeted anti-cancer drug delivery platform.

In recent years, dendrimers have become increasingly popular in the field of targeted drug delivery. Dendrimers are defined as synthetic macromolecules characterized by high branching points, three-dimensional globular shape, monodispersity, and nanometric size range. This surge in attention can be attributed to their unique and multivalent structure. Galactosylated dendrimers exhibit enhanced affinities towards specific receptors, allowing them to interact with specific cell types with greater selectivity and activity [43].

**Figure 5:**
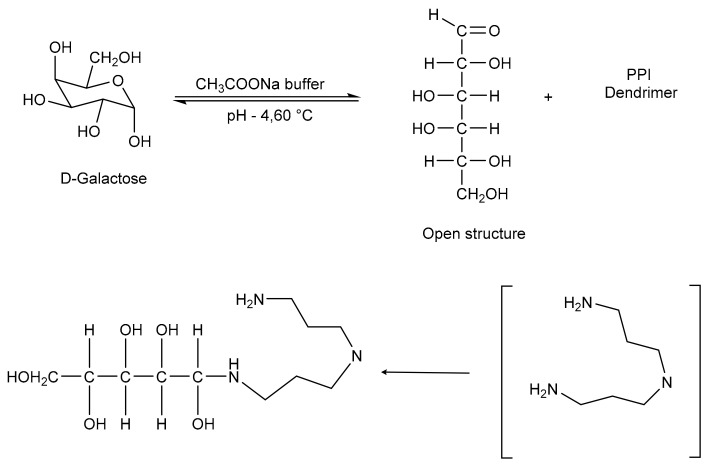
Synthesis of galactose-functionalized PPI dendrimers.

Kesharwani et al. conducted a study in which they investigated the cancer-targeting potential of galactose-anchored dendrimers [21]. As depicted in Figure 5, they synthesized Gal functionalized polypropyleneimine (PPI) dendrimers and encapsulated Paclitaxel (PTX). The efficacy of dendritic formulation was then evaluated in vitro using SiHa and HeLa cell lines. The results obtained from cellular uptake and cell viability assays demonstrated remarkable cytotoxicity of the galactose-modified formulations in both SiHa and HeLa cell lines.

**Scheme 6:**
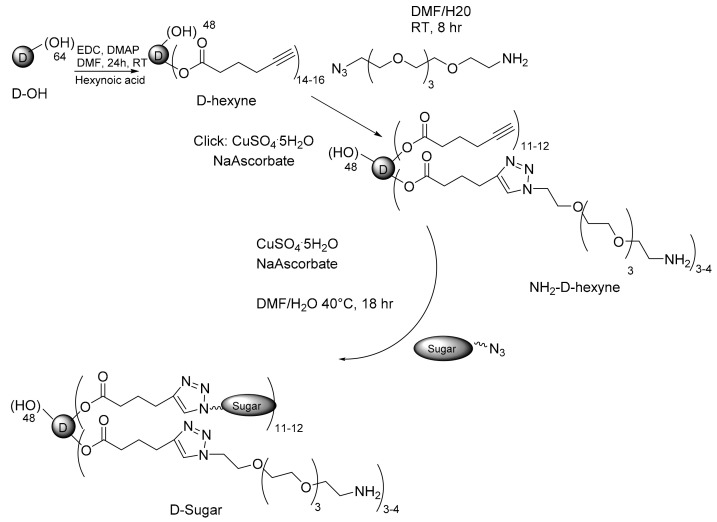
Synthesis of PAMAM generation 4 hydroxyl-terminated dendrimers with ethylenediamine-core (64 hydroxyl terminal-groups, D-OH).

A third-generation galactose-functionalized poly(propyl ether imine) (PETIM) dendrimer was synthesized and studied for its ability to interact with siRNA by Lakshminarayanan et al. [22]. The dendritic nano-vector, called DG, is designed to preferentially deliver siRNA to liver cells through a specific interaction between dendritic Gal and ASGP-R. The siRNA-DG complex exhibits perinuclear localization in liver cells and co-localization with viral proteins. Targeted delivery of siRNA helps to minimize adverse ‘off-target’ effects and maximize the efficacy of therapeutic response. In vitro and in vivo studies demonstrate the effectiveness of the siRNA-DG complex in inhibiting hepatitis C virus (HCV) replication. Also, poly(amidoamine) (PAMAM) dendrimers, with their unique branching structure and functional groups, have shown promise as drug delivery vehicles for glioblastoma, a highly aggressive and invasive form of brain cancer. PAMAM generation 4 hydroxyl-terminated dendrimers with ethylenediamine-core (64 hydroxyl terminal-groups, D-OH), whose synthesis is reported in Scheme 6, are able to cross the impaired blood–brain barrier upon systemic administration and selectively target activated microglia/macrophages in a variety of neurodegenerative diseases [44]. One of the challenges in treating glioblastoma is the ability to specifically target tumor cells while avoiding healthy brain tissue. Employing an efficient click chemistry method, Sharma et al. have precisely altered dendrimer surfaces by introducing sugar such as glucose, mannose, or Gal units (Scheme 6).

This modification aims to target overexpressed sugar transporters associated with glioblastoma. The attachment of Gal units to the dendrimer surface facilitates the engagement with galectins present on the membranes of glioblastoma cells, thereby facilitating targeted intervention in the tumor microenvironment [45].

PAMAM dendrimers have also been employed by other researchers. Ebeid and colleagues have described the synthesis of a liver-targeted gene delivery system using the galactose derivative lactobionic acid [23]. LA is conjugated to bifunctional poly(ethylene glycol), which is further conjugated to PAMAM. The conjugation of PEG, a widely used and safe hydrophilic polymer, to the surface of PAMAM avoids toxic interaction with the membranes of RBCs, which would cause hemolysis. The resulting PAMAM-PEG-LA construct can complex and deliver genetic material (e.g., pDNA, siRNA, mRNA) specifically to hepatocytes. The synthesis of PAMAM-PEG-LA is achieved using carbodiimide click chemistry. Next, nanoplexes are prepared by combining the positively charged conjugate and the negatively charged genetic material at different nitrogen-to-phosphate (N/P) ratios, and it seems to have the potential to serve as a universal gene delivery system to treat nonalcoholic steatohepatitis (NASH), as well as any other type of genetic abnormality that arises in hepatocytes.

For the NASH treatment, Sharma and coworkers have presented the rational design and development of a hepatocyte-targeting glycodendrimer called GAL-24 [24].

GAL-24 is constructed using biocompatible building blocks through a fast and straightforward chemical methodology. The unique design of GAL-24 allows it to specifically target the ASGP-R1 on hepatocytes, leading to its significant accumulation in the liver, with 20% of the injected dose detected just 1 h after systemic administration. Remarkably, GAL-24 demonstrates exceptional specificity for hepatocytes, as evidenced by more than 80% of hepatocytes exhibiting, after 24 h, a detectable signal of GAL-24−Cy5, which is a fluorescent labeling using cyanine 5 (Cy5). Furthermore, GAL-24−Cy5 retained its hepatocyte-targeting capabilities in both a mouse model of severe acetaminophen poisoning-induced hepatic necrosis and a rat model of NASH. These findings indicate the potential of the GAL-24 nanoplatform for enhanced drug delivery to hepatocytes, offering promising prospects for the treatment of various liver disorders. The next phase in this field will be designing and testing a GAL-24−drug conjugate. Significantly, GAL-24 eliminates the need for any subsequent modifications for the incorporation of a targeting ligand, making it inherently capable of targeting specific cells. Additionally, the total number of reaction steps required to synthesize GAL-24 is comparable to the processes involved in introducing targeting moieties on the surface of commercially available dendrimers.

### 2.2. Galactose Conjugated Directly to the Parent Drug

The use of galactosylated prodrugs has emerged as a highly effective strategy for improving the therapeutic potential of nonsteroidal anti-inflammatory drugs (NSAIDs), which are extensively utilized worldwide. Despite their widespread use, long-term exposure to NSAIDs can lead to serious cardiovascular events such as myocardial infarction and stroke, as well as gastrointestinal damage, including bleeding, ulceration, and perforation, which can be fatal. Additionally, kidney injury, hepatotoxicity, and other relatively minor adverse effects may occur [46].

To mitigate gastroduodenal injury, proton pump inhibitors (PPIs) are frequently co-prescribed. However, recent studies have demonstrated that the combination of PPIs with NSAIDs can exacerbate certain side effects of NSAIDs and induce additional complications such as dysbiosis [47].

**Scheme 7:**
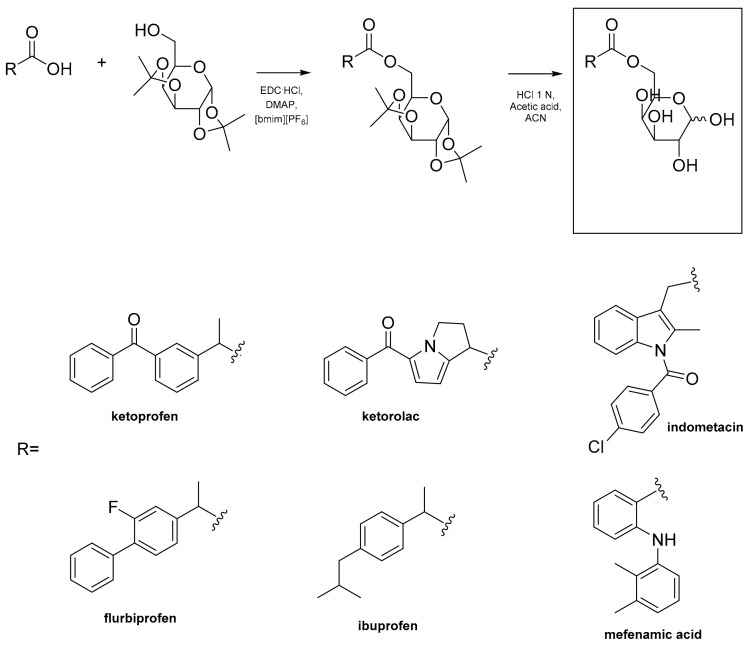
Synthesis of galactosylated NSAID prodrugs.

Exciting progress has been made in the field of prodrug development using Gal as a carrier for NSAIDs. In a study by Magliocca et al. in 2017, six galactosylated NSAID prodrugs (indomethacin, flurbiprofen, ketoprofen, mefenamic acid, ketorolac, and ibuprofen, named IndoGAL, FluGAL, OkiGAL, MefeGAL, KetoGAL, IbuGAL, respectively) were synthesized using both traditional and environmentally friendly methods (Scheme 7). The chemical and enzymatic stability of these prodrugs were assessed in vitro to facilitate subsequent in vivo pharmacological experiments [48].

The synthesis of galactosylated prodrugs involves the formation of an ester bond between the carboxylic acid of the parent drug and the hydroxyl group at the 6′ position of Gal. To achieve this conjugation, specific hydroxyl groups of the sugar are conveniently substituted, and subsequent cleavage reactions are performed to obtain the final prodrug. One effective approach for synthesizing esters is to utilize 1,2,3,4-di-*O*-isopropylidene-D-galactopyranose (DIPG), a commercially available galactosyl derivative that contains ketals serving as protective groups for other hydroxyl positions. Importantly, galactos-6′-yl ester prodrugs have demonstrated their utility in enhancing cell permeability and improving the pharmacokinetic properties of various drugs, thereby significantly reducing the occurrence of adverse effects while maintaining the efficacy of the parent drugs.

In another study published by the same group in 2021, the pharmacological and toxicological profiles of these galactosylated prodrugs were reported to be superior to those of the parent drugs, with a significant reduction in ulcerogenicity [25].

**Figure 6:**
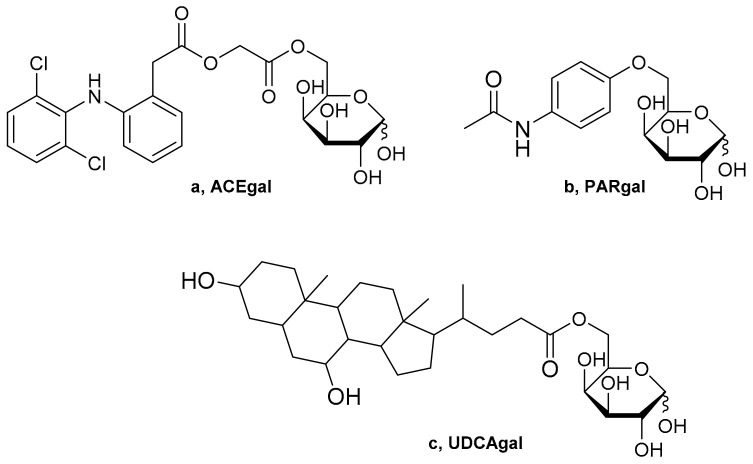
Chemical structures of galactosylated prodrugs of NSAIDs: ACEgal (**a**), PARgal (**b**), UDCAgal (**c**).

Equally impressive results have been achieved through the application of galactosylated prodrug technology to the analgesic and anti-inflammatory drug aceclofenac [26]. In a study conducted by an Italian research team, the stability, solubility, in vitro and in vivo cellular permeability of the ACEgal molecule were evaluated (Figure 6a). ACEgal demonstrated a hydrolysis half-life of 36 min in human serum, generating aceclofenac. Notably, ACEgal exhibited four times higher solubility in simulated gastric solution compared to aceclofenac and demonstrated equal intestinal permeability. ACEgal exhibited improved physicochemical, toxicological, and pharmacological profiles in comparison to aceclofenac, including a reduction in gastrointestinal toxicity, inflammation, analgesia, and oxidative stress. Furthermore, the anti-platelet aggregation activity was assessed.

ACEgal is not the sole example of an analgesic prodrug that has been developed. In a study by Sodano et al. (2019), the synthesis and characterization of a galactosylated prodrug of paracetamol (PARgal, Figure 6b) were described with the objective of enhancing the pharmacodynamic and pharmacological properties of paracetamol [27]. PARgal displayed favorable physicochemical properties, solubility, lipophilicity, and chemical stability at various physiological pH values and in human serum. Importantly, PARgal exhibited reduced hepatic metabolism in comparison to paracetamol and did not exhibit any cytotoxic effects in in vitro analyses conducted on human liver cells. Additionally, in an animal pain model, PARgal demonstrated a sustained analgesic effect for up to 12 h after oral administration.

Significant advancements have also been made in the development of galactosylated prodrug derived from the potent NSAID ketorolac. Recent studies by Russo et al. (2017) and Sodano et al. (2021) have demonstrated that Ketogal, the galactosylated prodrug of ketorolac, exhibits lower toxicity in the stomach, kidney, small intestine, and liver compared to ketorolac [28,29]. In vivo and ex vivo experiments conducted on mice have consistently shown that repeated administration of Ketogal induces reduced toxicity in these organs, supporting its potential as a safer alternative to ketorolac. Additionally, both drugs have shown similar therapeutic activity in an inflammation and pain perception animal model.

Another successful example concerns ursodeoxycholic acid (UDCA) and its galactosylated prodrug (UDCAgal, Figure 6c) [30]. UDCA is a bile acid that is used to treat cholestatic disorders, but UDCA has limited aqueous solubility, which can limit its bioavailability. To address this issue, it is necessary to synthesize UDCA prodrugs that are more soluble in water and can be more easily absorbed by the body. UDCAgal is designed to enhance not only solubility but also to exploit at the same time specific transporters in the liver. UDCAgal is quite stable and regenerates the parent drug after incubation in human plasma. Its solubility is higher than UDCA and is pH- and temperature-independent. UDCAgal has been shown to have a higher potency than UDCA in reducing serum biomarkers and cytokines in cholestatic rats. It also displayed a higher cell permeation compared to UDCA in liver HepG2 cells. These results indicate that UDCAgal has potential in the treatment of cholestatic disease.

Galactosylated prodrugs have been studied as a potential treatment for senescent cells, which accumulate during aging and are associated with various age-related pathologies. Senescent cells (SnCs) commonly exhibit higher levels of lysosomal β-galactosidase, an enzyme that breaks down the β-glycosidic bond between a Gal molecule and its organic component, which can be exploited as a marker for senescence [49,50]. This enzymatic activity can be utilized to create galactose-based prodrugs by chemically attaching Gal or acetyl Gal groups to a cytotoxic compound. These galactosylated prodrugs are preferentially processed in SnCs after being taken up by the cells, leading to the release of active cytotoxic agents and targeted elimination of SnCs while sparing non-senescent normal and proliferative cells. The effectiveness of this approach has been demonstrated through various galactose-based prodrugs.

**Figure 7:**
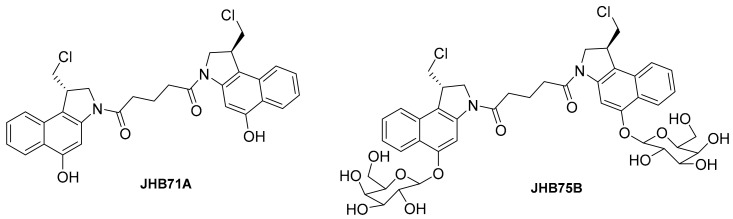
Chemical structures of JHB71A and JHB75B.

A galactose-modified duocarmycin prodrug (JHB75B, Figure 7) was developed by linking two Gal groups to a cytotoxic seco-duocarmycin analog dimer (JHB71A, Figure 7) [31]. Duocarmycin is a natural antibiotic and a highly cytostatic compound [51]. JHB75B can eliminate a broad range of senescent cells in culture and enhances the elimination of bystander senescent cells that accumulate upon whole-body irradiation or DOX treatment of mice. In a mouse model of human adamantinomatous craniopharyngioma (ACP), a pituitary pediatric tumor, treatment with JHB75B selectively reduced the number of β-catenin-positive preneoplastic senescent cells [32].

**Figure 8:**
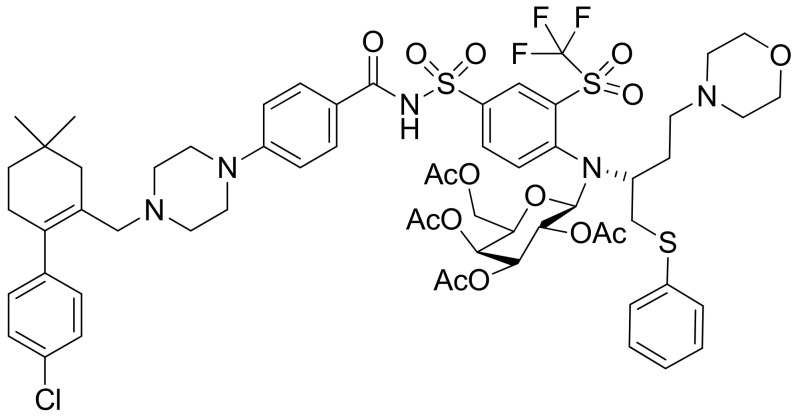
Chemical structure of Nav-Gal.

This prodrug approach has also been applied to reduce the significant hematological toxicity of navitoclax (Nav). A senolytic navitoclax prodrug (Nav-Gal, Figure 8) was generated by conjugating the BCL-2 family inhibitor navitoclax with an acetylated Gal group [33]. Nav-Gal showed effective senolytic activity in chemotherapy-induced senescent A549 lung cancer cells and SK-Mel-103 melanoma cells, with a higher senolytic index than navitoclax, which suggests that the pro-drug is preferentially activated in SnCs. Concomitant treatment with Nav-Gal and chemotherapeutic cisplatin significantly inhibited tumor growth in both subcutaneous tumor xenografts and orthotopic mouse models of chemotherapy-induced senescence in the context of lung carcinoma. Remarkably, compared to navitoclax, Nav-Gal treatment did not cause thrombocytopenia and showed reduced platelet toxicity in mice.

**Figure 9:**
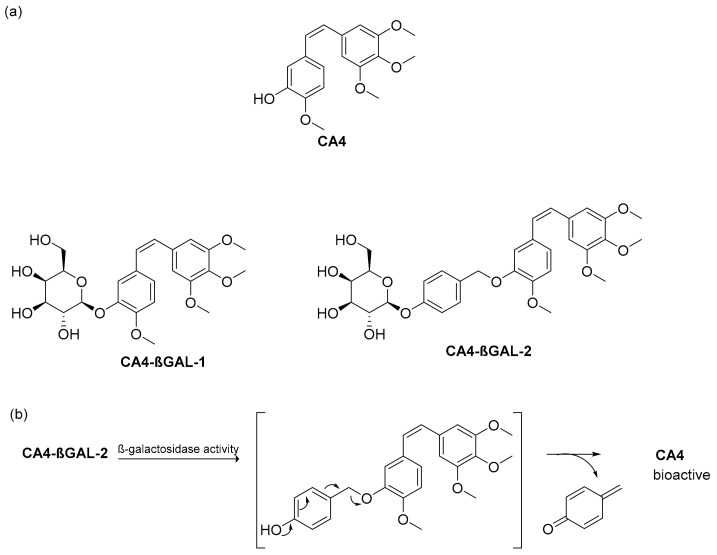
(**a**) Chemical structures of CA4, CA4-βGAL-1 and CA4-βGAL-2; (**b**) transformation of CA4-βGAL-2 into CA4 in response to β-galactosidase activity.

Doura and colleagues have investigated the conjugation of Combretastatin A4 (CA4) with a galactosyl moiety, resulting in Combretastatin A4-β-galactosyl conjugates for ovarian cancer prodrug monotherapy (PMT) [34]. CA4 (Figure 9a) is a natural compound known for its potent anti-cancer properties. However, its clinical application has been limited due to its poor solubility and rapid metabolism in the body. To address these challenges, the researchers have developed two CA4-βGal prodrugs, namely CA4-βGAL-1 and CA4-βGAL-2, that undergo a transformation into CA4 by releasing their β-GAL moieties in response to β-galactosidase activity (Figure 9a). In the case of CA4-βGAL-1, a glycoside bond directly introduces a β-GAL moiety to the hydroxy group on ring B. On the other hand, CA4-βGAL-2 utilizes a self-immolative benzyl linker inserted between CA4 and the β-GAL moiety. As shown in Figure 9b, the removal of the β-GAL moiety by β-galactosidase triggers 1,6-benzyl elimination of the linker, resulting in the formation of CA4. Previous studies have emphasized the significance of self-immolative linkers in prodrugs activated by a tumor-associated protease such as plasmin, as they help avoid steric hindrance near the tripeptide substrate moiety, enabling effective drug release. Hence, the researchers hypothesized that CA4-βGAL-1 would undergo slow metabolism by β-galactosidase due to the proximity of the methoxy group on ring B to the β-GAL moiety. In contrast, CA4-βGAL-2 would experience rapid decomposition by β-galactosidase owing to minimal steric hindrance near the β-GAL moiety. CA4-βGAL-2 exhibited more potent cytotoxicity to ovarian cancer cell lines than CA4-βGAL-1. This result indicates the suitability of introducing the self-immolative 1,6- benzyl linker in the design of prodrugs for ovarian cancer PMT. Because high β-galactosidase activity is reported in not only ovarian cancer but also in other malignant tumors, CA4-βGAL-2 can also be used as a prodrug candidate for PMT for these malignant tumors.

**Figure 10:**
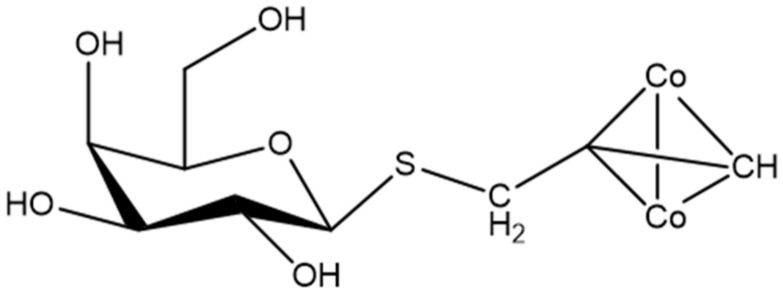
Chemical structure of complex 1.

A study synthesized carbon monoxide-releasing molecules (CORMs) based on carbohydrates that target Gal receptors and evaluated their anti-cancer activity [35]. Among CORMs, the complexes containing cobalt have various bioactivities, including anti-inflammatory and anti-tumor effects. These complexes have shown lower toxicity in animal tests, and cobalt does not accumulate in the body, but it is excreted through urine. Despite damaging the liver and kidney with consecutive administrations, CORMs based on cobalt have the potential to be used as medicine. The structure–activity relationship study suggests that the biodistribution of the complexes is influenced by side chain substituents, and alkyne substituents significantly affect CO-release rate, cytotoxicity, and cell viability. However, most CORMs currently available have poor solubility and lack selectivity for specific tissues and organs. To address these limitations, a series of CORMs based on carbohydrates, particularly Gal and sialic acid, were synthesized. Introducing Gal and sialic acid improved the solubility, activity, and selectivity of the CORMs towards target molecules. Complex 1 (see Figure 10) showed significant activity against various cancer cell lines and demonstrated better selectivity than the standard drug cisplatin (DDP). Moreover, the uptake ratio of complex 1 by specific cell lines was high, indicating its potential for targeted delivery. The mechanism of action of this complex involves cell cycle arrest at the G2/M phase, upregulation of caspase-3 and Bax (pro-apoptotic proteins), and downregulation of Bcl-2 (anti-apoptotic protein). Additionally, it reduces reactive oxygen species (ROS) and lowers mitochondrial membrane potential, similar to other cobalt-based CORMs reported previously. Complexes containing Gal or sialic acid showed improved solubility and selectivity towards target cells. However, further research is necessary to determine whether these complexes can be used as candidates in clinical settings.

## 3. Galactose for Diagnostics

The success of cancer treatments like surgery and endoscopy relies on quickly and accurately locating tumors and removing them completely. While larger tumors can be seen with the naked eye, tiny cancerous areas (less than 2 to 3 mm) are often invisible, leading to incomplete removal and increasing the chances of recurrence. Consequently, researchers are exploring the use of optical fluorescence-guided imaging to aid physicians during cancer surgeries. Fluorescent probes that can be activated only when they come into contact with the target tissue, triggered by specific conditions like enzymes (ex. β-Galactosidase), pH, or temperature, are being developed. This type of optical probe naturally produces fewer background signals, significantly improving the ratio between the target and the background (known as target-to-background ratio or TBR). One common approach to activate these optical probes involves the tumor microenvironment’s natural enzymatic activity, which is either absent in normal tissue or present in much lower levels. Enzyme-activatable optical probes can be applied topically through spraying during surgical and endoscopic procedures, enabling the identification of lesions and their boundaries for resection. Gal has been investigated as a broad ligand for multiple tumor imaging by conjugating to imaging agents, such as fluorescent dyes or radioactive tracers, to enable the visualization and detection of specific tissues or diseases. This can aid in the diagnosis, staging, and monitoring of various conditions. Gal residues can be specifically recognized by the ASGP-R, which is highly expressed on liver tissues. However, ASGP-R has not been widely investigated on different tumor cell lines except for hepatoma carcinoma cells, which motivated researchers to investigate the possibility of Gal serving as a broad tumor ligand. One study reported an approach for transforming an aggregation-induced emission (AIE) active molecule to a galactose-functionalized colloidal nanoparticle that can be used for targeting/labeling cancer cells [52]. The nanoparticles selectively label cells with overexpressed Gal receptors and may be used for cancer cell targeting and imaging.

In a study performed by Ma et al., a galactose-based probe conjugated with fluorescence dye MPA (GAL-MPA) was constructed to evaluate tumor affinities/targeted ability on different tumor cell lines [53]. The fluorescence probe GAL-MPA displayed higher cell affinity to tumor cells (HepG2, MCF-7, and A549) than to normal liver cells L02 in vitro. The in vivo dynamic study of GAL-MPA in tumor-bearing mice (HepG2, MCF-7, A549, HCT116, U87, MDA-MB-231, and S180) showed its high tumor-targeted ability with the maximal tumor/normal tissue ratio up to 6.8. Meanwhile, the fast tumor-targeted ability within 2 h and long retention on the tumor site up to 120 h were observed. These results demonstrated that Gal should be a promising broad ligand for multiple tumor imaging and targeted therapy.

## 4. Galactose for Theranostics

Gal has been investigated for its potential as a theranostic agent, although it is not commonly used for this purpose. Theranostics refers to the integration of therapy and diagnostics, where a single agent can perform both functions. As a theranostic agent, Gal can be utilized in several ways; for example, it can be attached to various chemotherapy drugs or nanoparticles to specifically target cancer cells that overexpress Gal receptors on their surface. This targeted approach aims to enhance the effectiveness of treatment while minimizing side effects on healthy tissues. Galactose-based theranostics is an active area of research, and researchers are exploring various approaches to develop safe and effective theranostic agents. Here, we want to highlight some recent developments in this field and how researchers are approaching the theranostic approach with Gal. By utilizing innovative materials and techniques, they aim to develop safe and effective methods for imaging, diagnostics, and targeted therapy in various biomedical applications. Despite significant advancements in cancer diagnosis and treatment, the field of oncology, which tackles one of the primary causes of global mortality, continues to demand innovative therapies that can enhance effectiveness while minimizing the adverse effects associated with traditional treatments.

Highly fluorescent CdSe/CdS quantum rods were synthesized for specific biolabeling and controlled drug release. To enhance biocompatibility, the nanocrystals were conjugated with PEG and enveloped in a carbohydrate shell, facilitating recognition by GLUT-1. Additionally, dopamine was attached through an ester bond, enabling controlled drug release via hydrolysis by esterases. The CdSe/CdS quantum rods served as smart nanotools for targeted biolabeling and controlled drug release. Moreover, Malvindi et al. utilized galactose-coated CdSe/CdS quantum rods to release dopamine [54]. They conjugated succinyl dopamine to the Gal shell using the EDC (1-etil-3 (3-dimetilaminopropil) carbodiimide) crosslinking method. Upon cellular uptake, the conjugate was readily recognized and hydrolyzed by esterases, leading to dopamine release.

**Figure 11:**
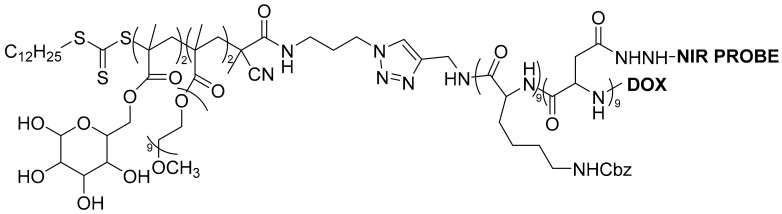
Chemical structure of a galactose-targeted pH-responsive amphiphilic multiblock copolymer conjugated with DOX and a NIR fluorescent probe.

Fu et al. designed a galactose-targeted pH-responsive amphiphilic multiblock copolymer conjugated with a drug (DOX) and a near-infrared (NIR) fluorescence probe for imaging of intelligent drug delivery [55]. The study found that the galactose-targeted polymeric prodrug (see Figure 11) showed a fast and enhanced endocytosis due to specific interaction with HepG2 cells, indicating that the polymer could be a candidate for theranosis of liver cancer.

**Figure 12:**
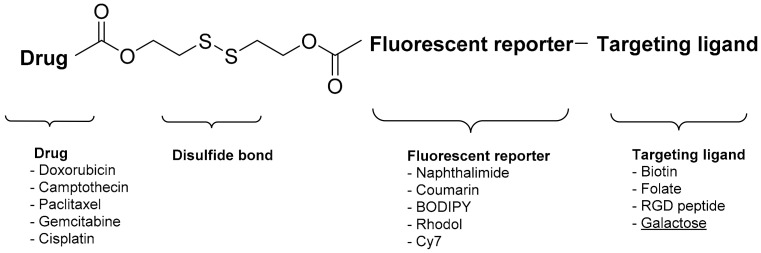
Schematic illustration of disulfide-based fluorescent drug delivery conjugates.

In another paper published by Lee and co-workers, disulfide-based fluorescent drug delivery conjugates were investigated, including preliminary tests to assess their biological utility both in vitro and in vivo [56]. Disulfide bonds are particularly attractive for the construction of theranostic conjugates because they are stable in most blood pools but are efficiently cleaved by cellular thiols, including glutathione and thioredoxin (Trx), which are generally found at elevated levels in tumors. Disulfide-based multifunctional conjugates are multicomponent constructs that selectively target cancer cells and deliver cytotoxic agents while producing a readily detectable signal that can be monitored both in vitro and in vivo. These conjugates require the synthesis of relatively complex systems comprising imaging reporters, masked chemotherapeutic drugs, cleavable linkers, and cancer-targeting ligands. A variety of chemotherapeutic agents and fluorophores have been used to create disulfide-based conjugates, including DOX, gemcitabine, camptothecin, naphthalimide, coumarin, BODIPY, rhodol, and Cy7, as shown schematically in Figure 12.

In the context of liver diseases, galactose-based theranostics were also explored to deliver therapies directly to the liver or to detect liver abnormalities through imaging techniques. Quan et al. prepared galactose-based nanogels to facilitate the targeted delivery of iodoazomycin arabinofuranoside (IAZA), a clinical drug for imaging solid hypoxic tumors, and evaluate its role in hypoxia-selective (radio)theranostic management of therapy-resistant cancer cells [57]. The nanogels have a crosslinked temperature-responsive core and a dense carbohydrate shell. Their thermoresponsive nature allowed the controlled encapsulation of the IAZA drug for targeted delivery and release in hypoxic hepatocellular carcinoma via ASGP-R-mediated uptake.

**Figure 13:**
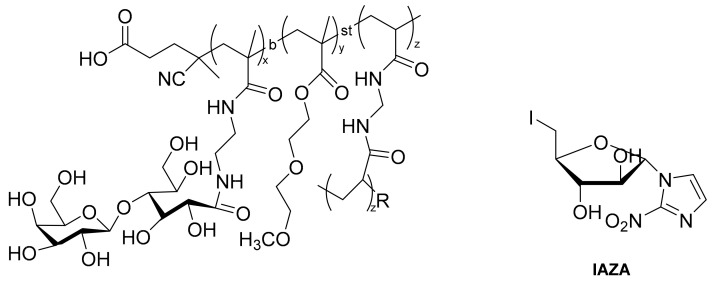
Chemical structures of IAZA and galactose-based nanogel.

The optimization of nanogels mentioned above was conducted by Diaz-Dussan in a recently published paper [58]. The study focuses on the development of a hypoxia-directed nanosensitizer formulation for the treatment of solid tumors. The formulation consists of a nanogel composed of a galactose-based shell and a thermoresponsive di(ethylene glycol) methyl ethyl methacrylate core. The main objective was to overcome the poor vascularization of solid tumors, which hinders oxygen supply and drug delivery to the cells. By encapsulating IAZA, a hypoxia-activated prodrug (HAP), within the galactose-based nanogel (Figure 13), the researchers aimed to enhance the sensitivity of hypoxic tumors to the administered drug. The optimized nanogel formulation has the potential to improve the treatment of hypoxic tumors by addressing their specific needs.

Several new targets and approaches are being evaluated for ovarian cancer targeting with an emphasis on improving detection and applications in image-guided surgery [59]. Nearly 50% of primary ovarian cancers show enhanced enzymatic activities of β-galactosidase compared to normal ovaries, and it has been a focus of enzymatically activated fluorescence probe development to visualize ovarian cancer metastases. NIR photoimmunotherapy (NIR-PIT) is a therapeutic approach that combines the specificity of targeted immunotherapy with the cytotoxic effects of light-based therapy. It involves the use of NIR light and a photosensitizer to selectively destroy cancer cells while sparing surrounding healthy tissues. NIR-PIT studies often use antibodies as the targeting moiety. A non-antibody-derived NIR-PIT agent was developed by Harada et al. and tested in a disseminated ovarian cancer model. Galactosyl serum albumin (GSA), which is composed of a Gal molecule conjugated to albumin via carboxyl groups, was used. This construct can bind to the β-D-Gal receptor, a surface lectin, which is overexpressed in many cancers, including ovarian cancers. β-D-Gal receptor is quickly internalized after binding to ligands. In the study, the researchers aimed to evaluate the efficacy of NIR-PIT using GSA as a targeted photosensitizer in this model. The general principle of NIR-PIT involves the administration of the photosensitizer followed by the application of NIR light. The NIR light activates the photosensitizer, leading to the generation of cytotoxic reactive oxygen species that induce cell death. By selectively targeting cancer cells with GSA and then applying NIR light, the researchers aimed to achieve localized destruction of the diffuse peritoneal disseminated ovarian cancer cells while minimizing damage to healthy tissues. The study likely involved in vitro experiments using cancer cell lines, as well as in vivo experiments using animal models of diffuse peritoneal disseminated ovarian cancer. In particular, the agent GSA-IR700 accumulated specifically in the tumor, and repeated regimens of NIR-PIT improved the treatment efficacy by increasing the depth of GSA-IR700 delivery into tumor nodules. The study showed specific delivery of GSA-IR700 to the tumor and reduction of the metastatic burden after NIR-PIT. The results of this study would provide insights into the potential of NIR-PIT with galactosyl serum albumin as a treatment strategy for diffuse peritoneal disseminated ovarian cancer and could contribute to the development of more effective therapies for this challenging condition.

**Figure 14:**
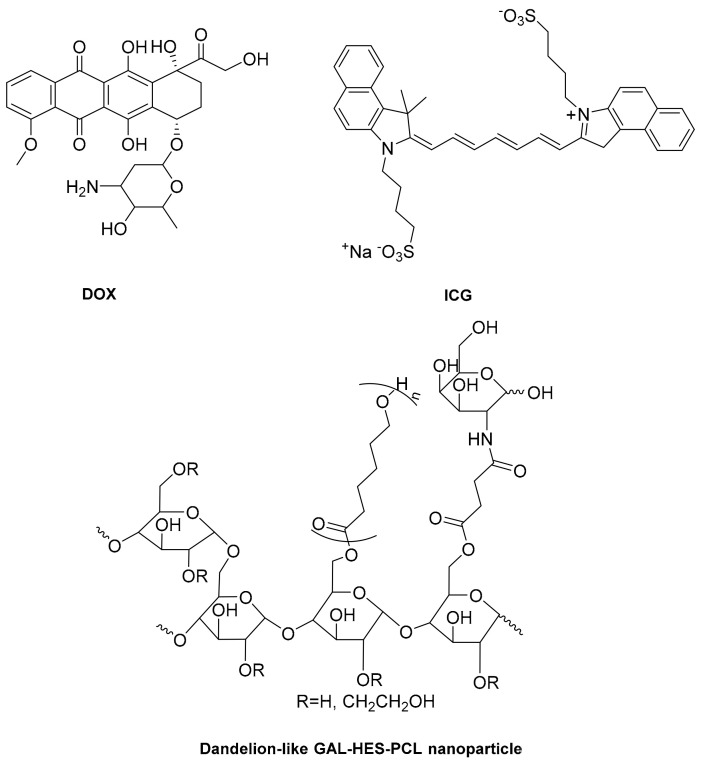
Chemical structures of DOX, ICG, and DOX/ICG-GAL-HES-PCL NCs.

One study proposed the use of nanocolloidosomes for active tumor-targeted imaging-guided photothermal/chemo combination therapy [60]. The idea was to use the nanocolloidosomes to deliver both a chemotherapy drug and a photothermal agent to the tumor site and then use near-infrared light to trigger the release of the drugs and heat up the tumor cells, causing them to die. Nanocolloidosomes are a type of nanoparticle that can be used for targeted drug delivery. They consist of a lipid bilayer that encapsulates a hydrophilic core and can be functionalized with targeting ligands to selectively bind to tumor cells. The nanocolloidosomes can be designed to selectively release the drugs in response to specific stimuli, such as changes in pH or temperature, to minimize off-target effects. This approach has the potential to improve the efficacy of cancer treatment while reducing side effects. Hu et al. created, for the first time, novel multifunctional theranostic nanoplatforms called DOX and indocyanine green (DOX/ICG)-loaded nanocolloidosomes (NCs) to enable photothermal/chemo combination therapy. These NCs were designed with selective drug release capabilities. They were fabricated through a templating process using galactose-functionalized hydroxyethyl starch-polycaprolactone (GAL-HES-PCL) nanoparticles-stabilized Pickering emulsions. The resulting DOX/ICG-GAL-HES-PCL NCs (see Figure 14) had a diameter of approximately 140 nm. They exhibited remarkable tumor-targeting ability, effective tumor penetration, and enhanced photothermal effects. Furthermore, these NCs were capable of NIR fluorescence imaging, allowing real-time imaging of solid tumors with excellent contrast. In comparison to DOX/ICG-HES-PCL NCs and a DOX/ICG mixture, these NCs demonstrated superior in vivo anti-tumor efficacy when combined with laser irradiation. As a result, these NCs hold great value for active tumor-targeted imaging-guided combination therapy against liver cancer and potentially other diseases.

**Scheme 8:**
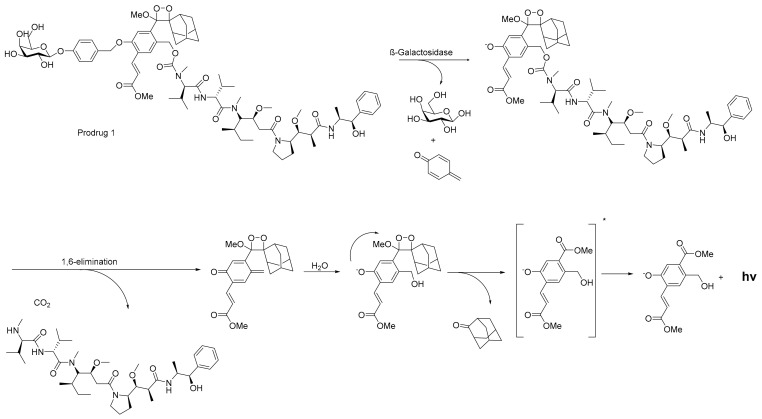
Mechanism of activation of prodrug 1.

β-Galactosidase is often overexpressed in various tumor types and has been utilized as an active trigger for tumor-targeting strategies [61]. In a study by Gnaim et al., the first theranostic prodrug called 1, monitored by a chemoluminescence diagnostic mode, was designed and synthesized [62]. Prodrug 1 consisted of three components: a cytotoxic agent called monomethyl auristatin E (MMAE), a tumor-specific response unit β-Gal, and a reporter molecule called phenoxy dioxetane. The activation of prodrug 1 was initiated by enzymatic removal of the β-Gal unit, triggering a series of reactions that resulted in the emission of green photons, as reported in Scheme 8. This allowed for the visualization of tumor cells with elevated levels of β-galactosidase. The chemiluminescence detection mode exhibited significant advantages over other theranostic agents that employed fluorescence imaging, primarily due to its superior signal-to-noise ratio.

Furthermore, prodrug 1 demonstrated a 20-fold lower IC_50_ in HEK293 cells that stably expressed β-galactosidase compared to wild-type HEK293 cells (with IC_50_ of 1 nM and 20 nM, respectively). This finding confirmed the targeted anti-tumor efficacy of prodrug 1. The results of Gnaim et al.’s study indicated that the chemiluminescence diagnostic mode could be a promising approach for designing theranostic agents and facilitating personalized tumor treatment. 

**Figure 15:**
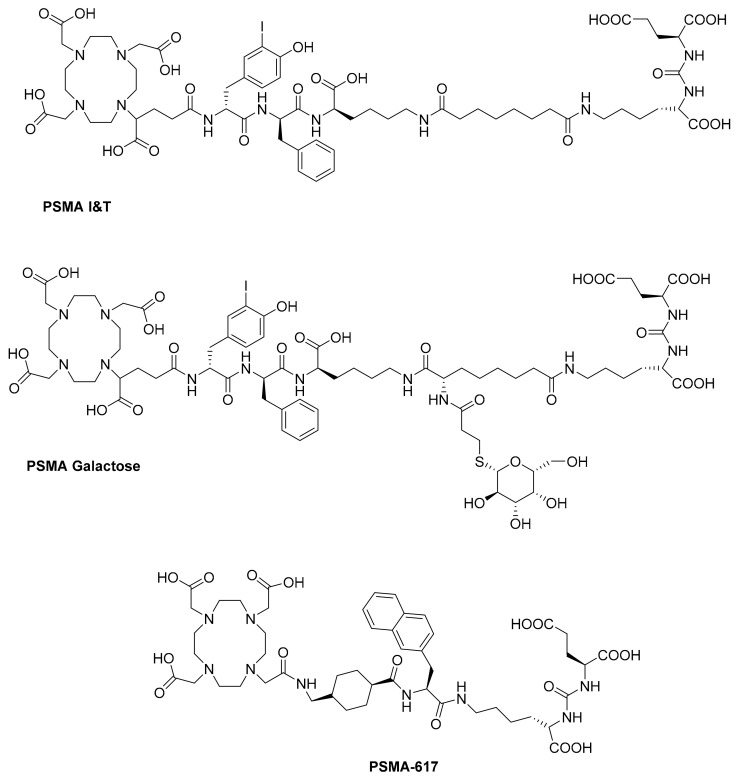
Chemical structures of PSMA I&T, PMSA Galactose, and PSMA-617.

Another study investigated the effect of carbohydration on the theranostic tracer PSMA I&T (see structure in Figure 15), which is used for prostate-specific membrane antigen imaging and therapy [63]. The discovery of prostate-specific membrane antigen (PSMA), a type II transmembrane protein, revealed its significant overexpression on a majority of prostate cancer (PCa) cells. Notably, as the disease progresses, the level of PSMA expression increases. The exceptional characteristics of PSMA as a target have facilitated the development of radiolabeled PSMA inhibitors and antibodies, which are used for imaging and endoradiotherapy specifically for prostate cancer. The researchers aimed to understand how the addition of carbohydrate moieties, referred to as PSMA Galactose (Figure 15), impacts the pharmacokinetic profile of PSMA inhibitors. It was found that carbohydration had minimal effect on the lipophilicity of the tracer but did decrease PSMA-mediated internalization. The binding of the tracer to human serum albumin (HSA) was significantly reduced with the carbohydrated derivative compared to the original PSMA I&T (Figure 15). In small-animal positron emission tomography (μPET) imaging, the carbohydrated derivative showed lower nonspecific tissue and kidney accumulation than the original tracer but slightly lower tumor uptake as well. Biodistribution studies confirmed reduced nonspecific uptake in non-target tissues and decreased renal accumulation of the carbohydrated derivative, resulting in improved tumor-to-tissue ratios overall. However, carbohydration did not have a significant positive effect on the targeting performance of PSMA I&T in vivo. Despite this, carbohydration expands the possibilities for modifications within the linker area and could be a valuable tool for the future development of PSMA inhibitors with reduced nonspecific tissue accumulation and improved pharmacokinetics.

Currently, a phase 3 clinical trial is evaluating [^177^Lu] (lutetium) PSMA I&T for the treatment of metastatic castration-resistant prostate cancer. On March 23, 2022, the United States Food and Drug Administration (FDA) approved Pluvicto (^177^Lu 177 vipivotide tetraxetan, also known as ^177^Lu-PSMA-617 or PSMA-617) for the treatment of adult patients with PSMA-positive metastatic castration-resistant prostate cancer [64].

**Scheme 9:**
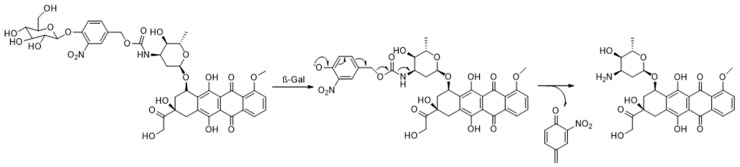
Mechanism of action of GAL-DOX.

Sharma et al. developed a Gal moiety-modified prodrug of DOX, and this prodrug could serve as an excellent targeting ligand for ASGP-R, as well as β-galactosidase activated drug with the help of the additional Gal moiety [65]. A small molecule-based theranostic system, GAL-DOX, is preferentially taken up by colon cancer cells through receptor-mediated endocytosis. Once inside the cancer cell, the GAL-DOX complex is released from the endosome and enters the cytoplasm, as reported in Scheme 9. DOX molecule, which is a potent chemotherapeutic agent, is then free to exert its cytotoxic effects on the cancer cell, ultimately leading to cell death. Additionally, GAL-DOX can also serve as a theranostic system, combining therapy and diagnostic capabilities. This is achieved by incorporating a fluorescent or radioactive label into the GAL-DOX complex. The label allows for non-invasive imaging techniques such as fluorescence imaging or positron emission tomography (PET) to visualize the accumulation of GAL-DOX in colon cancer cells, providing information about the tumor location and response to therapy. The use of GAL-DOX as a theranostic system offers several advantages. First, it provides targeted delivery of the chemotherapeutic agent, minimizing off-target effects and reducing toxicity to healthy cells. Second, the imaging component allows for real-time monitoring of the treatment efficacy, enabling adjustments in therapy if necessary. Overall, GAL-DOX shows promise as a targeted theranostic approach for colon cancer treatment.

In a recently published paper, Dang et al. describe the development of a nanoprobe that can be used for in situ NO release monitoring and NO prodrug delivery [66]. The probe is a galactose-modified benzothiadiazole-based fluorescent probe (GalNONP/C) that exhibits far-red emission in the range from 550 to 800 nm and has an acidity preference. Gal on the probe enables selective targeting of hepatocellular carcinoma cells by binding to the ASGP-R on the cell surface. The probe also delivers low-molecular weight NO prodrug JS-K into cells and monitors the real-time release of the generated NO. In vivo NO imaging with tumor targeting was demonstrated in HCC orthotopic transplantation nude mice and liver sections. Compared with the control experiment using a probe without NO prodrug loading, a higher fluorescence response of NO was detected in the cell (3.0 times) and liver slices of the HCC tumor model (2.7 times). This strategy may pave the way to develop nanoprobes for in situ NO monitoring and therapy evaluation in NO-related cancer therapy.

**Figure 16:**
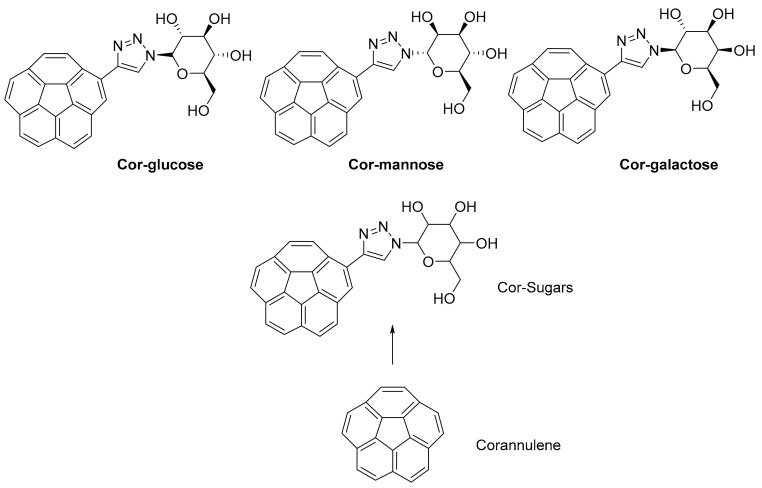
Chemical structures of Cor-sugars.

Liu et al. have investigated a new category of anti-cancer agents called corranulene monoglycoconjugates (Cor-sugars, Figure 16) [67]. They explored various aspects of these compounds, including their synthesis, cytotoxicity, effectiveness in vivo, molecular uptake, and distinctive mechanisms for targeting tumors. The design approach behind conjugating monosaccharides with corannulene via a triazolyl moiety was motivated by the following objectives: (1) enhancing water solubility through glycoconjugation to demonstrate the physiological and pharmacological properties associated with a single molecule that remains unaggregated, (2) improving the fluorescent characteristics of corannulene by incorporating a triazolyl π-system to enable luminescence-based self-probing both in vitro and in vivo, (3) investigating the potential of extensively conjugated π-systems of Cor-sugars to interact with DNA in cancer therapy, and (4) utilizing the high expression of glucose transporters (GLUTs) in cancer cells (known as the Warburg effect) to achieve selective uptake of the compounds in tumors. Corannulene triazolyl monosaccharides, including galactose-conjugated Cor-gal (Figure 16), were found to exhibit highly potent cytotoxicity against human cancer cells and effectively inhibit xenograft growth of hyperglycolytic tumors. Cor-gal exhibited superior in vivo anti-cancer efficacy in A549 tumor models with an outstanding safety profile compared to DOX. The study also revealed that Cor-sugars exploit tumor-specific glucose transporter glucose transporter 1 (GLUT1) for targeted cell delivery and intra-tumoral accumulation through the cancer-specific Warburg effect.

Recently, there has been a growing interest in utilizing 2D materials for targeted drug delivery due to their unique properties and large surface area [68]. One such material, 2D molybdenum disulphide (MoS_2_), has shown biocompatibility and minimal toxicity to healthy cells. However, the development of carbohydrate-modified MoS_2_ materials for targeted delivery of anti-cancer agents has been challenging.

**Figure 17:**
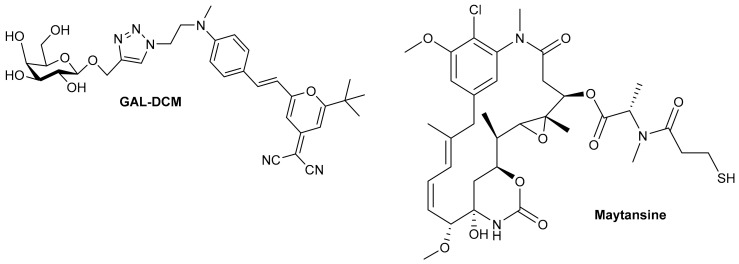
Chemical structures of GAL-DCM and Maytansine.

In this study, the researchers focused on creating a multicomponent “glycoprotein” by incorporating a fluorescent glycoprobe galactosyl dicyanomethylene-4H-pyran (GAL-DCM, Figure 17) and an anti-cancer drug (Maytansine, Figure 17) into the hydrophobic pockets of HSA. HSA is a protein abundantly found in the human body and can serve as a supramolecular host for various diagnostic and therapeutic probes, offering enhanced properties.

The researchers demonstrated that they could construct supramolecular “glycoproteins” suitable for biomedical applications by including a dye-modified glycoside into the hydrophobic cavity of HSA. They further assembled these glycoproteins with 2D MoS_2_2 to create a 2D glycomaterial.

The 2D glycomaterial showed the ability to selectively deliver therapeutic cargo to Hep-G2 cells, which express an ASGP-R on their surface, leading to cell apoptosis. The researchers achieved this through host–guest interactions between a galactosyl dye and HSA, resulting in supramolecular galactoside-HSA conjugates that coated the 2D MoS_2_. The study demonstrated that the 2D glycomaterial successfully targeted the delivery of Maytansine to a liver cancer cell line expressing the Gal receptor. This targeted delivery approach resulted in greater cytotoxicity compared to using Maytansine alone. In summary, this study aimed to develop a carbohydrate-modified 2D MoS_2_ material for targeted delivery of anti-cancer agents. By incorporating a fluorescent glycoprobe and an anti-cancer drug into HSA and assembling it with 2D MoS_2_, they created a 2D glycomaterial capable of selectively delivering therapeutic cargo to cancer cells expressing a Gal receptor. The results showed enhanced cytotoxicity, highlighting the potential of this approach for improved therapeutic outcomes.

**Scheme 10:**
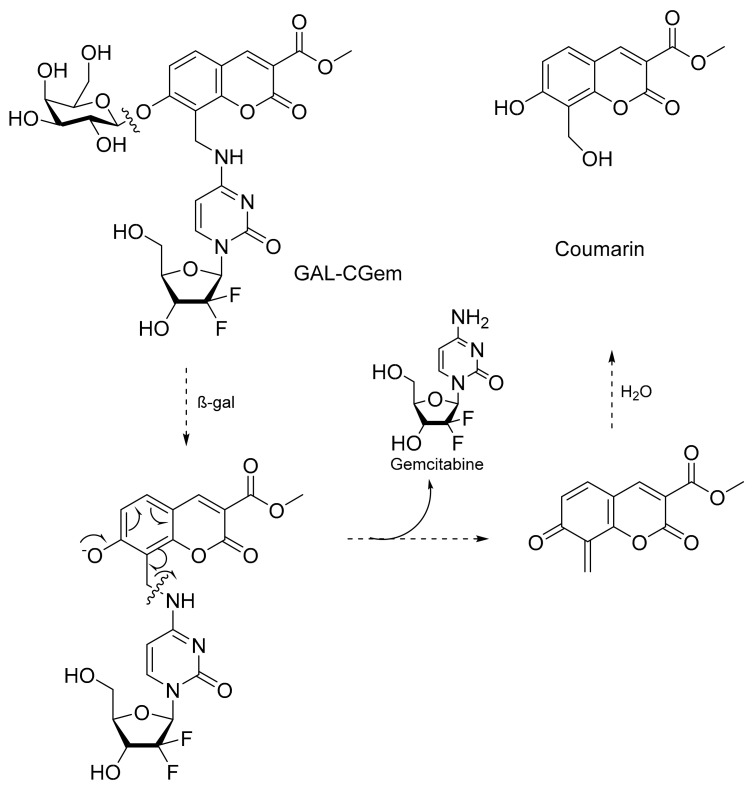
Mechanism of action of GAL-CGem.

As previously said, the targeting of β-galactosidase in cancer cells has emerged as a promising strategy for the development of β-galactosidase-activated anti-cancer prodrugs. Different works have focused on β-galactosidase-activated theranostics using DOX as a self-reporting agent. Among them, Maiti et al. [69] have developed a β-galactosidase-activated theranostic based on gemcitabine called GAL-CGem (Scheme 10). GAL-CGem consists of a galactose-substituted coumarin scaffold linked to gemcitabine, an anti-cancer drug.

The β-galactosidase enzyme, which is overexpressed in cancer cells, cleaves the β-GAL from coumarin, leading to an intramolecular rearrangement that releases gemcitabine as depicted in Scheme 10. This release also generates a fluorescent coumarin derivative, allowing for monitoring gemcitabine release. Gemcitabine is known to have a short intracellular half-life, typically ranging from 8 to 94 min. However, this strategy extended the lifetime of gemcitabine in the cellular environment, thereby increasing its anti-cancer activity. This study establishes GAL-CGem as a promising theranostic system that is selectively activated in cancer cells with high levels of β-galactosidase while sparing neighboring healthy cells from adverse effects.

In another study, the primary objective was to create two types of micelles for targeted delivery to HepG2 cells: GAL-PEG-b-PLA targeted micelles and allyl PEG-b-PLA non-targeted micelles [70]. These micelles were designed to encapsulate within their hydrophobic core both a superparamagnetic iron oxide (SPIO) contrast agent and a hydrophobic drug called RSPP050, which possesses anti-cancer activity. Fluorescence imaging and Prussian blue staining were performed to evaluate the cellular uptake of RSPP050 and SPIO. The results showed that galactose-targeted SPIO micelles efficiently delivered both RSPP050 and SPIO into HepG2 cells. At the 3-h time point, galactose-targeted micelles exhibited significant cellular uptake and iron uptake compared to non-targeted micelles. Additionally, magnetic resonance imaging (MRI) data indicated that the T2-weighted image intensity of galactose-targeted SPIO micelles was lower than that of non-targeted SPIO micelles after 3 h. These experiments confirmed the effectiveness of the theranostic function of GAL-RSPP050/SPIO micelles. The positive outcomes laid the foundation for future in vivo studies.

## 5. Conclusions

Gal has proven to be a versatile and promising carrier in drug delivery, offering numerous advantages such as enhanced targeting, improved bioavailability, and reduced systemic toxicity.

In addition to drug delivery, galactose-based carriers have shown great potential in diagnostic applications. The ability of Gal to specifically bind to certain receptors overexpressed in diseased cells has allowed for the development of targeted imaging agents and diagnostic tools. These advancements have paved the way for early detection and accurate diagnosis of various diseases.

The emerging field of theranostics, which combines therapy and diagnostics, has benefited greatly from the utilization of Gal as a carrier. Galactose-based carriers can be loaded with therapeutic agents while simultaneously carrying imaging agents, enabling real-time monitoring of treatment efficacy. This synergistic approach holds great promise for personalized medicine and improving patient outcomes.

Over the past decade, researchers have made significant strides in optimizing the design and functionality of galactose-based carriers. The development of multifunctional carriers that can encapsulate multiple drugs, imaging agents, and targeting ligands has expanded the scope of galactose-mediated drug delivery, diagnostics, and theranostics.

Despite the considerable progress, challenges still remain in the field of galactose-based carriers. Further studies are needed to address issues related to stability, scalability, and manufacturing processes. Additionally, understanding the long-term effects and potential side effects of galactose-based carriers is crucial for their successful translation into clinical applications.

Looking ahead, the continued exploration of Gal as a carrier in drug delivery, diagnostics, and theranostics holds immense potential. The integration of galactose-based carriers with emerging technologies, such as nanotechnology and molecular imaging, will likely lead to groundbreaking advancements in the field of precision medicine, targeted therapies, and personalized medicine.

## Figures and Tables

**Table 1:** Examples of galactose conjugated to drug carriers reported in the present review.

Drug Carrier	Active Moiety	Targeted Organ	Targeting Receptor	In Vitro Studies	In Vivo Studies	Ref.
PHEA-EDA-DPPE-GAL	RBV tripalmitate	Liver	ASGP-Rs	HepG2	-	[11]
PHEA-EDA-PLA-GAL	RBV tripalmitate	Liver	ASGP-Rs	HepG2	-	[12]
AG-PLG	RMP	Liver	ASGP-Rs	HepG2	Female ICR mice	[13]
GC	FUA	Liver	ASGP-Rs	HepG2 and A549	Sprague–Dawley rats	[14]
GC-FUA	miRNA-122	Liver	ASGP-Rs	HepG2	BALB/c nude mice	[15]
GC-FUA	FA	Liver	ASGP-Rs and FRs	SMMC-7721	BALB/c nude mice	[16]
GAL-disulfide linker sensitive to GSH	W-105	Liver	ASGP-Rs	HepG2	H22 tumor-bearing mice	[17]
PEG-Sulfo-BSA-GAL	H_2_S Prodrug	Liver	ASGP-Rs	HepG2 and RAW264.7	Mice	[18]
P(GAL-b-DPA-b-AzMA)	Alkynyl-JSK	Liver	ASGP-Rs	HepG2	-	[19]
GAL-P5	CPT-SS-PEG	Liver	ASGP-Rs	HepG2 and HeLa	-	[20]
GAL-conjugated PPI dendrimers	PTX	-	-	SiHa and HeLa	-	[21]
DG	siRNA	Liver	ASGP-Rs	Liver cells	Mice	[22]
PAMAM-PEG-LA	Genetic material	-	-	-	-	[23]
GAL-24	-	Liver	ASGP-Rs	HepG2	Mice and NASH mouse model	[24]

**Table 2:** Examples of galactose conjugated directly to parent drugs reported in the present review.

Galactosylated Prodrugs	In Vitro Studies	In Vivo Studies	Ex Vivo Studies	Ref.
IbuGAL and OkiGAL and IndoGAL and FluGAL	Serum protein binding	Carrageenan-induced hyperalgesia Acetic Acid-induced Writing Test Ulcerogenic activity	Intestinal Permeation	[25]
ACEgal	Intestinal Permeability	Carrageenan-induced hyperalgesia Acetic Acid-induced Writing Test Ulcerogenic activity	Inhibition of platelet aggregation	[26]
PARgal	HepG2	Post-operative Pain model Mechanical Hypergesia	-	[27]
Ketogal	-	Mechanical Hypergesia Thermal Hypergesia Carrageenan-induced hyperalgesia	Histological evaluation of Intestine, Stomach, Kidney, Liver	[28,29]
UDCAgal	Liver Permeation	EE-induced cholestasis	Analysis of bile flow	[30]
JHB75B	Senescent cells	ACP model	-	[31,32]
Nav-Gal	A549 and SK-Mel-103	Tumor xenografts and orthotopic models	-	[33]
CA4-βGAL-1 and CA4-βGAL-2	Ovarian Cancer cells	-	-	[34]
Complex 1	HeLa and HepG2 and MCF-7 and HT-29	-	-	[35]

## Data Availability

Not applicable.
